# Combined Molecular Algorithms for the Generation, Equilibration and Topological Analysis of Entangled Polymers: Methodology and Performance

**DOI:** 10.3390/ijms10115054

**Published:** 2009-11-23

**Authors:** Nikos Ch. Karayiannis, Martin Kröger

**Affiliations:** 1 Institute for Optoelectronics and Microsystems (ISOM) and ETSII, Universidad Politécnica de Madrid (UPM), José Gutiérrez Abascal 2, E-28006 Madrid, Spain; 2 Polymer Physics, Swiss Federal Institute of Technology, ETH Zurich, Wolfgang-Pauli-Strasse 10, 8049 Zurich, Switzerland

**Keywords:** polymer, simulation, Monte Carlo, primitive path, knot, entanglement, random packing, hard-sphere, melt, system size effect, polydispersity

## Abstract

We review the methodology, algorithmic implementation and performance characteristics of a hierarchical modeling scheme for the generation, equilibration and topological analysis of polymer systems at various levels of molecular description: from atomistic polyethylene samples to random packings of freely-jointed chains of tangent hard spheres of uniform size. Our analysis focuses on hitherto less discussed algorithmic details of the implementation of both, the Monte Carlo (MC) procedure for the system generation and equilibration, and a postprocessing step, where we identify the underlying topological structure of the simulated systems in the form of primitive paths. In order to demonstrate our arguments, we study how molecular length and packing density (volume fraction) affect the performance of the MC scheme built around chain-connectivity altering moves. In parallel, we quantify the effect of finite system size, of polydispersity, and of the definition of the number of entanglements (and related entanglement molecular weight) on the results about the primitive path network. Along these lines we approve main concepts which had been previously proposed in the literature.

## 1. Introduction

During the last decades numerous and foremost advances in various technological areas constantly render computer processors faster and more powerful at an affordable cost allowing the formation of super-computers of even myriads of processors. In parallel, scientific breakthroughs in the various fields of computer simulations have lead to the development of novel algorithms that are capable of taking full advantage of the allocated resources. Thus, it is not surprising that nowadays modeling and simulations are widely accepted as valuable companions to the more “mature” experimental and theoretical studies. Computer-generated specimen of varied molecular detail and diverse chemical constitution can be simulated under “idealized” and well-controlled conditions addressing “what-if” questions on the structure-property relation that otherwise would require the execution of a series of cost-demanding and time-consuming experiments. However, severe limitations often hinder the effectiveness and plague the performance of conventional simulation techniques necessitating the fabrication of novel algorithms or even their combined employment through hierarchical and multi-scale modeling schemes.

**Figure 1.**
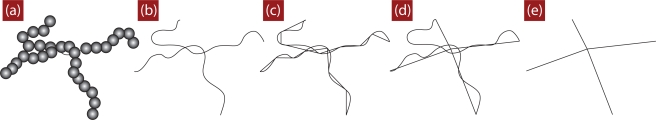
Schematic drawing summarizing the construction of the (three–dimensional) primitive paths (PP). (a) Microscopic configuration. For clarity reasons, two out of hundreds of chains are shown. (b) For the construction of the PP within the Z1 code, the backbone is considered infinitely thin, and chain ends are fixed in space. (c) The length of the multiple disconnected path is monotonically reduced, subject to chain-uncrossability, by introducing a smaller number of nodes. (d) Upon iterating the geometrical procedure one converges at a final state, the shortest path, shown in (d), together with the original chain, and alone in (e). Each chain carries a part of the multiple disconnected shortest path, called its PP. A single PP is often characterized by its conformational properties such as reflected by contour length *L*_pp_ and number of kinks, denoted as *Z*. Self–entanglements are not taken into account into the analysis.

In particular, regarding macromolecular (polymer) systems the complex chemical constitution of the monomer (repeat) units along with the large spectrum of characteristic length and time scales require the development and employment of highly-efficient, system-specific methodological approaches. For example, in a typical polymer melt the shortest characteristic distance is that of the bond length *l* (*O*(10^0^) Å). For conceptual purposes a chain can be further divided into equivalent freely-jointed Kuhn segments of uniform size. For flexible chain molecules each segment typically spans up to a dozen of successive monomers [1]. Chain size for a molecule of *N* repeat units is generally quantified by the mean square end-to-end distance *〈R*^2^*〉* or the mean square radius of gyration $\langle R_{g}^{2}\rangle$. It is well established that in the asymptotic limit of very long chains (*N → ∞*) the two quantities are interrelated through the Debye equation for a random walk [2]: $\langle R^{2}\rangle \;=\;6\langle R_{g}^{2}\rangle$. Polymer stiffness is more suitably expressed by the characteristic ratio, *C_N_* a measure that incorporates chemical and molecular details of the constituent monomers. At infinite chain length it stands *C_∞_* = *〈R*^2^*〉 /*(*N –* 1)*l*^2^ [3]. Experiments have shown that for a linear polyethylene (PE) melt of average molecular length C_1000_ (where the number in the subscript denotes the carbon atoms along the chain) the average end-to-end distance *〈R*^2^*〉*^0.5^ is by almost two orders of magnitude higher than the carbon-carbon bond length (*l ≃* 1.54Å) [4]. Similarly to the diversity of lengths scales, the relaxation spectrum of polymer melts spans from the fastest dynamical mode corresponding to bond vibration – on the order of femtoseconds, *i.e.*, *O*(10^−15^) s – moving to the de-correlation time of torsional (dihedral) angles, usually on the order of picoseconds, *i.e.*, *O*(10^−12^) s [5,6] – up to the slowest global chain relaxation. The latter has been quantitatively described by the Rouse [7] and reptation [8,9] models for “relatively short” and “adequately long” chain molecules, respectively. According to the tube model and the reptation theory the sluggish chain motion is strongly hindered by topological constraints (entanglements) imposed by surrounding macromolecules. Thus, the motion of the inner chain segments is restricted along the contour path of the tube; perpendicular motion is set only for short time scales and for distances that at most commensurate the tube radius. The axis of the tube constitutes the primitive path (PP) defined as the shortest path that connects the two ends of the chain by fully respecting all imposed topological constraints, cf. Figure 1. According to numerous experimental studies for well entangled polymer melts at equilibrium the diffusivity of the center of mass, *D*_G_, and the corresponding zero shear-rate viscosity, *η*_0_, scale with molecular length *N* as *D*_G_ *∼ N^−b^* and *η*_0_ *∼ N^c^*, respectively, with *b ≃* 2.3 and *c ∼* 3.4 (see for example [10,11] and references within). Zero-shear rate viscosity is proportional to the reptation time *τ_d_* which is defined as the characteristic time needed for the whole primitive chain to escape the original confining tube [9]. For a linear C_1000_ PE melt, *τ_d_* is on the order of tens of microseconds (*O*(10^−5^) s)), which is about ten orders of magnitude longer than the vibrational relaxation of bonds. The terminal relaxation mechanism is even more complicated for polymers exhibiting highly non-linear chain architecture like long-chain branching, stars or rings [12–16].

Building on the original tube model by de Gennes [8,17,18] and Doi-Edwards [9] a large body of theoretical studies and predictive models, often accompanied by supporting experiments, provided significant insights into the molecular mechanisms that govern the dynamical and rheological behavior of complex polymeric systems (see for example Refs. [19–34]). From the modeling perspective for polymer systems the large spectrum of characteristic time and length scales along with the diversity of the chemical detail of the repeat units require the development of advanced case-specific simulation techniques. In parallel, different length scales necessitate the employment of varied methods that span from quantum mechanical calculations to atomistic and more meso/macroscopic simulations. Consequently, to establish a (micro)structure - (macro)property relation so as to address typical, industrially-relevant polymer processes and phenomena one has to resort to multiscale, hierarchical approaches that combine the aforementioned techniques and exchange data between different levels of system representation [35–40]. Regarding atomistic simulations a wealth of information on the static and dynamic properties of polymers melts of low to intermediate average molecular weight (MW) can be extracted from conventional Molecular Dynamics (MD) [41–43] simulations. MD is the most straightforward and attractively general computational method since in its core implementation it solves the classical (Newton’s) equations of motion for a set of molecules (particles) [41]. However, for long and definitely entangled polymer systems it is well established that the equilibration afforded by atomistic MD simulations, especially regarding the long-range characteristics, is inadequate even when highly-sophisticated, state-of-the-art realizations [44,45] are employed in massively parallel simulations. Since the fastest relaxation time (that of bond length) of chain molecules imposes a time integration step that can not exceed a couple of femtoseconds, months or even years of computational time are required for MD to reach times on the order of tens of microseconds which correspond to the chain reptation time of entangled polymer melts (see discussion above).

The challenge of polymer equilibration can be addressed by mapping the atomistic reference system into a less-detailed “coarse-grained” configuration which is characterized by significantly fewer degrees of freedom allowing for simulations which extend over time and lengths scales that are not accessible in the atomistic level of description. Numerous schemes exist for the systematic “coarse-graining” of the monomer units of various polymers resulting into supersites (*i.e.*, segments of atoms lumped into single interacting sites) that range from spherical blobs to complex ellipsoids [46–54]. In recent modeling approaches equilibration is afforded through a fine-graining scheme which is performed on initial coarse-grained polymer representations to produce well-relaxed atomistic ones [55].

Alternatively, the equilibration of macromolecular systems can be achieved through stochastic Monte Carlo (MC) [41–43] simulations where trial moves are attempted between two configurational states and are accepted or rejected according to the Metropolis criterion. In the last two decades many “smart” MC techniques (moves) such as reptation [56], configurational bias [57–59], concerted rotation [60] and parallel rotation [61] have appeared for the simulation of dense polymer systems either for lattice-based or continuum simulations of varied chemical representation. Even the combination of such efficient algorithms is not able to provide robust sampling of the configurational space for truly polymeric substances of high MW. Towards this direction an advanced set of highly-sophisticated chain-connectivity altering moves (CCAMs) was developed in the last 15 years. These algorithms, through unphysical but “smart” transitions that entail appropriate re-arrangements of the connectivity of chains, provide a vigorous sampling of dense phases for a variety of polymer systems. Prominent among the CCAMs are the end-bridging (EB) [62,63] and the double-bridging (DB) [64,65] moves along with their intramolecular counterparts [64,65], variants [66] and similar algorithmic schemes [67–70]. In the past MC simulations based on CCAMs along with localized moves have successfully equilibrated, within modest computational time, in atomistic detail the short- and long- range structural characteristics and volumetric properties of polydisperse (up to C_6000_ [71]) and monodisperse (up to C_1000_ [64,65]) linear, H-shaped [72], short-chain branched [73] and star-like [74] polyethylene melts. The EB and DB algorithms have been further modified and expanded to treat atomistic systems of macromolecules with varied chemical constitution of the repeat units [75–77]. While MC simulations, by evolving as long sequences of stochastic processes, can not provide any information regarding the evolution of the system in time they do impart a wealth of information regarding the static, thermodynamic and volumetric properties of polymer melts and glasses. Furthermore, through hierarchical modeling schemes, MC-based trajectories can be used as well-equilibrated starting configurations for successive modeling studies, to extract for example information on the dynamics (through extensive MD simulations [78–80]) or on the barrier properties (through Transition State Theory (TST) calculations [81]) of the simulated system.

We should note that the efficient application of MC schemes, especially the ones built around CCAMs, is not straightforward for chemically complex atomistic macromolecular systems where it may happen that the performance of the underlying moves is so poor that the whole MC scheme is rendered practically useless. For example, the ability of MC mixtures, based on the configurational bias algorithm [57–59], to provide full-scale equilibration for PE melts at ambient conditions declines sharply as the system chemical description transits from the united-atom (UA) to the explicit-atom (EA, where interactions of hydrogen atoms are taken into account) representation [82]. Solution to this and similar problems related to the “ruggedness” of the energy landscape of explicitly detailed molecular models can be provided by casting the aforementioned MC moves in a minimum-to-minimum mapping (Min-Map) sampling pattern [83] based on the reversible bijective method [84,85].

During the last years well equilibrated, representative polymer configurations obtained by different methods and at diverse levels of molecular detail have been subjected to extensive topological analyses for the extraction of the primitive paths (PP) and the corresponding network of intermolecular topological constraints (entanglements). The original algorithms for the construction of the PP network were based on secant areas [86] or energy minimizations through annealings [87–100] while latter topological approaches adopt either direct geometric (the “Z1” method [101,102]) or stochastic (the “CReTA” method [103,104]) algorithms for the identification of entanglements and for the calculation of the statistics of the corresponding PP network [52,53,101–112]. Through the primitive path analysis significant insights can be gained for the rheological behavior of polymer melts and solutions since the calculated quantities (for example the contour length of the primitive path, *L*_pp_) can be readily correlated with key parameters of the reptation theory [9] and of the tube model [8]. Furthermore, results obtained from the topological analysis on well equilibrated polymer samples [105] can validate newly-introduced analytical predictions [113,114], proposed formulas and expressions [109] regarding the behavior of PP-related statistics at and beyond equilibrium.

In the present contribution we review the salient technical characteristics of a new MC scheme [115], based on advanced chain-connectivity-altering moves, for the efficient generation and equilibration of random packings of athermal polymer models of freely-jointed chains of tangent hard spheres and its combination with the latest implementation of the Z1 algorithm [101,102] for the extraction of the underlying primitive path network. The paper is organized as follows: Section 2 presents the algorithmic details and reports on the equilibration efficiency of the MC method, while section 3 analyzes the effect of system size on the MC-based estimation of chain size. Section 4 focuses on the description of the Z1 algorithm along with the benchmark times required for the construction of the PP network. Section 5 summarizes the key features of the proposed hierarchical approach and of the underlying methodologies and concludes with proposed plans for future extensions and generalizations.

## 2. Monte Carlo Scheme for the Generation and Relaxation of Athermal Polymer Models

In the present section we will describe how the existing chain-connectivity altering moves [62–65,116], originally formulated for the atomistic simulation of polyethylene melts, can be modified so as to function with optimal efficiency in simulations of random packings of freely-jointed chains of tangent hard spheres of uniform size at packing densities (volume fractions), that range from dilute up to very dense ones. According to the freely-jointed chain model of hard spheres, there is no bending or torsional hindrance and pairs of spheres interact solely through the typical hard-core potential form, *U*(*r*) of:

(1) $$U(r)\;=\;\{\begin{array}{rrr} 0 & , & r\;\geq \;\sigma \\ \infty & , & r\;<\;\sigma \end{array}$$

where *r* is the distance between the spheres and *σ* is the sphere diameter (collision distance) being equal to the bond length for strictly tangent spheres. In the continuation, volume fraction (packing density) is defined as:

(2) $$\varphi \;=\;\frac{\pi n\sigma^{3}}{6V}$$

where *V* is the volume of the simulation cell and *n* is the total number of spheres in the system. In typical simulations of the bulk phase periodic boundary conditions are applied in all dimensions of the cell. The nature of the hard-core potential (Equation 1) allows for the decomposition of the original simulation cell into a number of equivalent sub-cells each with dimensions slightly larger than the collision distance *σ*. As a consequence, once a sphere-site (or a sequence of sites) is displaced during a MC move checks for hard-sphere overlaps are performed only with unmoved sites belonging to the closest neighboring cells of the new (trial) position.

In the original end-bridging (EB) algorithm [62] the move proceeds by cutting (removing) a properly selected trimer of atoms from the backbone of a chain (*jch*) that lies within a specific radius from the end of another chain (*ich*) and by re-connecting the trimer to the end of chain *ich* so as to form two new macromolecules with completely different connectivities. The DB algorithm [64,65] evolves around the re-construction of two trimer bridges, while in the intramolecular variants (IEB [116] and IDR [64,65,116]) the re-bridging(s) take place within a single chain. In general, all atomistic chain-connectivity altering moves rely on the following geometrical problem for the construction of the trimer bridge: “*two dimers in a three-dimensional space should be connected through a triplet so as the resulting heptamer bears prescribed bond lengths and bending angles*”. The original problem was solved through the psi-function formulation [62,63,65] while in a more recent approach for the MC simulation of cyclic peptides Wu and Deem mapped the problem into solving a sixteenth degree polynomial of inverse kinematics involving serial chain manipulators in robotics [117]. By construction, since after a successful EB move one of the participating chains (the “predator” *ich*) grows while the other (the “prey” *jch*) shrinks, polydispersity in molecular lengths is introduced in the simulated system. The form and the width of the chain length distribution can be fully controlled by casting the simulations in the semi-grand canonical statistical ensemble where the following parameters are held fixed: the total number of chains (*N*_ch_) and spheres (*n*), the pressure (*P*), the temperature (*T*) and the spectrum of chemical potentials μ* for all chain species expect two which are taken as reference. More details about the expressions of the chemical potentials required to reproduce commonly observed chain length distributions can be found in Ref. [62].

Double bridging (DB) alleviates the disadvantages and limitations of the EB move (mainly the requirements of chain length polydispersity and the presence of chain-ends) by performing two trimer bridgings in backbone segments that lie in the internal parts of the participating chains. Consequently, it is generally applicable to simulations of macromolecular systems with no free ends (cyclic peptides), with long branches (H-shaped macromolecules) or with specific constrained geometries (terminally-grafted brushes) [64]. Furthermore, since segments in DB are removed from and reconstructed in both participating chains, specific combinations can lead to chains with exactly the same molecular length as the original ones before the attempted move preserving monodispersity in molecular weights [64,65]. However, the major advantages that enhance the general applicability of DB come at a high cost: since two triplets of interacting sites are required to be repositioned in the system, the acceptance rate of DB is quite lower than the corresponding one of EB. For example, in simulations of monodisperse C_1000_ PE melts at *T* = 450K and *P* = 1atm approximately one move gets accepted over 80,000 trials [65], while the analogous acceptance rate of EB for polydisperse systems of the same average molecular length and under the same simulation conditions is around 0.2%.

The non-negligible deterioration of the acceptance rate for the class of chain-connectivity altering moves is especially apparent in simulations of dense packings of freely-jointed, hard-sphere chains primarily as a consequence of the reduced free volume available for the reconstruction of a whole trimer of hard spheres (or of two trimers for DB). Consequently, in the original implementation of the EB algorithm for dense systems of hard-sphere chains, for the vast majority of the attempted moves the reconstruction of the triplet of spheres of all candidate geometrical configurations leads to energetic overlaps with the fixed sites. A second source for the poor performance of chain-connectivity altering moves is that for freely-jointed chains the absence of any kind of imposed bending hindrance in the form of a potential function allows for the bond angles to fluctuate uniformly in the closed interval [0,120°] (except for the existence of characteristic peaks at high volume fractions as a result of the excluded volume interactions [115,118–120]). In turn, the geometric bridging of the two dimers with the triplet of spheres entails the random selection of five bending angles, that can not connect the dimers if they lie far apart, but are still within the maximum allowed bridgeable distance of 4*σ*. The aforementioned disadvantages and limitations can be removed by properly modifying the original chain-connectivity altering algorithms so as to function efficiently for any kind of force field that allows for close proximity (tangency) of intermolecular pairs of interacting sites. Towards this direction we have recently introduced the simplified end-bridging (sEB [115]) and its intramolecular variant termed simplified intramolecular end-bridging (sIEB [115]).

### 2.1. Simplified End-Bridging

**Figure 2.**
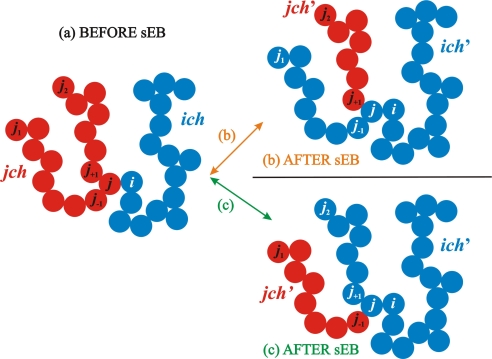
Schematic representation of the simplified end-bridging (sEB) move [115]. Left: initial configurations where chain end *i* of chain *ich* (shown in blue) is tangent (within a numerical tolerance) to intermolecular neighbor *j* that lies in the inner segments of chain *jch* (shown in red). Right: sEB proceeds by cutting the bond between sphere *j* and either sphere *j*_+1_ (pattern (b)) or sphere *j_–_*_1_ (pattern (c)). A new bond is formed between sites *i* and *j* altering the backbone connectivities and forming new chains *ich*′ and *jch*′. By construction, after a successful sEB move the number of monomers in chains *ich*′ and *jch*′ grows and shrinks, respectively, introducing polydispersity in molecular lengths.

The schematic representation of the sEB move is shown in Figure 2. The move is initiated as follows: an end-site *i* (the “predator”) of chain *ich* and an internal sphere *j* (the “prey”) of chain *jch* are tangent within a numerical tolerance of *dσ*, *i.e.*, the distance between spheres *i* and *j* is within the range [*σ*,*σ* + *dσ*]. A bond is temporarily formed between spheres *i* and *j* and depending on the molecular lengths of the resulting chains, sEB can proceed in at most two different combinations: in pattern (b) the bond between spheres *j* and *j*_+1_ is cut so that the whole sequence (*j*_1_*, ..., j*) that was originally part of *jch* belongs now to new chain *ich′* while new *jch′* consists of the remaining segment (*j*_+1_*, ..., j*_2_). In pattern (c) the bond between spheres *j* and *j_−_*_1_ is removed leaving the sequence (*j*_1_,...*j_−_*_1_) as *jch′* and connecting the part (*j*_2_*, ... j*) with the original *ich*. It is obvious that in both patterns (b) and (c) chain *ich* increases in molecular length while *jch* shrinks so that the total number of repeat units before and after the move remains the same. An inspection of Figure 2 shows clearly that the initial and final configurations of the two participating chains are very different. Furthermore, a remarkable feature of the sEB move is that while this alternation of molecular conformations is very profound it does not imply any sphere displacement, rather it proceeds by a single bond deletion and a consequent bond insertion. Accordingly, the transition between the initial (old) and final (new) states does not evolve any potential changes in system energetics.

In the final step of its application, the sEB move is accepted or rejected according to the following criterion:

(3) $$P_{\text{sEB}}(\text{old}\;\to \;\text{new})\;=\;\text{min}\;\left[1,\;\frac{w_{\text{sEB}}(\text{new}\;\to \;\text{old})}{w_{\text{sEB}}(\text{old}\;\to \;\text{new})}\right]$$

where *w*_sEB_ denotes the weighting factor for the attempted transition according to:

(4) $$w_{\text{sEB}}(\text{new}\;\to \;\text{old})\;=\;\frac{1}{n_{\text{sEB}}(j_{\text{end}})},\;\;\;\;\;\;\;\;w_{\text{sEB}}(\text{old}\;\to \;\text{new})\;=\;\frac{1}{n_{\text{sEB}}(i)}$$

*n*_sEB_ being the number of the neighbors with whom spheres *j*_end_ and *i* can initiate a sEB move in the final (new) and initial (old) states, respectively. Depending on the sEB combination the newly formed chain-end *j*_end_ of *jch′* corresponds to either site *j*_+1_ (pattern (b)) or to site *j*_−1_ (pattern (c)).

### 2.2. Simplified Intramolecular End-Bridging

**Figure 3.**
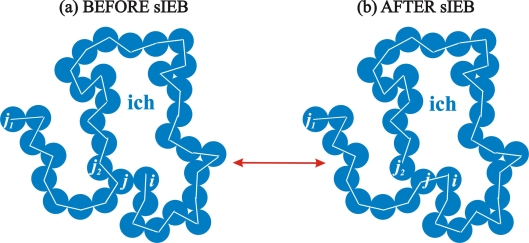
Schematic representation of the simplified intramolecular end-bridging (sIEB) move [115]. Left: initial configuration where chain end *i* of chain *ich* (with sequence (*j*_1_*, ..., i*)) is tangent (within a numerical tolerance) to intramolecular neighbor *j*. Right: sIEB proceeds by cutting the bond between spheres *j* and *j*_2_ while a new bond is formed between sites *i* and *j* altering the backbone connectivity of chain *ich* to sequence (*j*_1_*, ...j*_2_). By construction, a successful sIEB move does not alter the molecular length of *ich* but only its monomer sequence. The sequence of bonds along the backbone of the chain before and after the move is shown as a guide of the eye to illustrate the alternation of chain connectivity as a result of the bond swapping.

The simplified intramolecular end-bridging (sIEB) move, by being the intramolecular variant of the sEB algorithm, is executed in a very similar fashion, the only difference being that all alternations occur in a single chain. The schematic representation of sIEB is depicted in Figure 3. Chain *ich* consists of the original sequence of spheres (*j*_1_*, ..., i*). If chain-end *i* and internal site *j* lie within the bonded distance of *σ* + *dσ* (*i.e.*, they are tangent within the specified numerical tolerance of bond lengths) an sIEB move can be initiated. For any given pair of intramolecular pairs sIEB proceeds in a single pattern: the bond between sites *j* and *j*_2_ breaks and a new one is formed between *i* and *j*. As a result the sequence of spheres in *ich* changes to (*j*_1_*, ..., j*_2_) (as seen in Figure 3) and sphere *j*_2_ becomes the new terminal site of chain *ich*.

The acceptance criterion of the sIEB move is very similar to the corresponding one of sEB of Equation 3:

(5) $$P_{\text{sIEB}}(\text{old}\;\to \;\text{new})\;=\;\text{min}\;\left[1,\frac{w_{\text{sIEB}}(\text{new}\;\to \;\text{old})}{w_{\text{sIEB}}(\text{old}\;\to \;\text{new})}\right]$$

where now the weighting factors in the forward and reverse transitions can be calculated as:

(6) $$w_{\text{sIEB}}(\text{new}\;\to \;\text{old})\;=\;\frac{1}{n_{\text{sIEB}}(j_{2})},\;\;\;\;\;\;\;\;\;\;\;\;\;\;\;w_{\text{sIEB}}(\text{old}\;\to \;\text{new})=\;\frac{1}{n_{\text{sIEB}}(i)}$$

with *n*_sIEB_(*j*_2_) and *n*_sIEB_(*i*) being the number of intramolecular neighbors within the range of *σ* + *dσ*of end-spheres *j*_2_ and *i* in the reverse (new *→* old) and forward (old *→* new) transitions, respectively.

### 2.3. Algorithmic Implementation of the sEB and sIEB Algorithms

In the course of a typical MC simulation based on the chain-connectivity altering moves special lists are kept and are constantly updated for both sEB and sIEB. For every chain-end *i* present in the system distance checks are performed with all intra- and inter- molecular neighbors that reside in the closest sub-cells (the parent one where the reference end belongs to and the 26 closest neighboring cells, see Section 2. for more details on the division of the simulation box to equal sub-cells). If the distance between *i* and one of the neighboring sites *j* is smaller than *σ* + *dσ* then: i) if *j* is an intramolecular neighbor no further action is required and *i* and *j* are automatically added in the list of site pairs that can initiate an sIEB move, ii) if *j* belongs to another chain *jch*, the provisional molecular lengths of *ich′* and *jch′* are calculated based on the position index of *j* in *jch* for both patterns (b) and (c) of sEB (see Figure 2). In the case that, for at least one of the possible combinations ((b) and (c)), the new chain lengths of *ich′* and *jch′* both fall within the range of the allowed chain lengths, as imposed by the applied distribution, then the pair *i* and *j* is added in the corresponding list of sEB initiators along with the feasible combinations.

Both moves commence by selecting randomly one of the pairs included in their special lists. If an sEB (or sIEB) is attempted and its list of initiators is empty then the move is automatically rejected. For sEB if both combinations (b) and (c) are permitted then one is randomly picked. In the reverse transition (new *→* old) the (sEB or sIEB) neighbors of the new temporary chain end are counted since their number enters into the weighting factor of the reverse transition (Equations 4 and 6). Finally, the move is accepted or rejected according to the corresponding acceptance criterion, Equations 3 and 5 for sEB and sIEB, respectively. In case of acceptance of an sEB move, because of the major alternations in chain connectivities, the sEB-related lists are calculated from scratch for all chains in the system, while the ones of sIEB are computed only for *ich* and *jch*. In an analogous fashion, after an accepted sIEB move the sEB-related lists are re-calculated for all molecules but the sIEB-related ones are updated only for chain *ich*.

### 2.4. Monte Carlo Scheme Based on sEB and sIEB

The Monte Carlo scheme used for the simulation of the freely-jointed chains of tangent hard spheres is built around the sEB and sIEB moves and it further consists of local moves that undertake the task of providing short-range relaxation. Additionally, by combining chain-connectivity altering algorithms and a set of varied localized ones we are able to significantly reduce the “shuttling effect”, *i.e.*, when the system transits between a limited collection of different states ending up in the original one. For example, it is quite frequent for the system to perform, through a chain-connectivity altering move, the (old *→* new) transition and successively to annihilate it by moving back to the original state through the reverse move (new *→* old). As a consequence of the transitional loop (old *→* new, new *→* old) the system remains trapped in the original state. In order to significantly reduce or even eliminate the “shuttling effect” the special lists maintained for each move should contain as many as possible pairs of initiators and these should be frequently replaced by new ones. Towards this, efficient local algorithms, by moving a single site or a whole sequence of them either in the end of the chain or in the internal parts, provide the necessary invigoration in the special lists of sEB and sIEB. In the present MC mixture we employ the following localized moves: i) reptation [56], ii) intermolecular reptation [72,108,116], iii) end-mer rotation [41], iv) internal flip [121] and v) continuum configurational bias (CCB)) [57–59,122]. The concerted rotation (ConRot) move [60] was excluded from the current MC scheme since, in a fashion analogous to the original chain-connectivity altering algorithms, it entails the trimer bridging construction whose geometric solution becomes inefficient for freely-jointed chains (see also related discussion in Section 2.). A key characteristic in the application of the local algorithms in simulations of dense packings of hard-sphere chains is that they are all cast in an adaptive configurational-bias pattern with the number of the trial configurations *n*_dis_ in the reverse and forward transitions being strongly dependent on the volume fraction (packing density) *φ* [115]. For example, *n*_dis_ is equal to 10 and 80 at *φ* = 0.10 and 0.60, respectively. This increment of *n*_dis_ with *φ* guarantees the highest achievable acceptance rate of the move (see for example Figure 3 of [115]) without resorting to more complicated algorithmic approaches like casting all local moves in a bijective Min-Map bias pattern according to Ref. [83]. As a side effect, by increasing the number of trial configurations the average CPU time required per move grows too. Thus, the optimum value of *n*_dis_ is identified through preliminary trial simulations at the specified volume fraction.

The simulation of dense random packings of freely-jointed chains of tangent hard spheres splits into two phases: the first step of the generation for the simulation box filled with the non-overlapping chain monomers at the desired volume fraction *φ* and the second of the full-scale equilibration at constant density. The numerical challenge of generating long trajectories of random assemblies of monomeric hard spheres is readily addressed by various techniques [123–125] very close to and even at the random close packing (RCP) limit [126–128]. Nowadays the RCP limit tends to be replaced by the mathematically more firm concept of the maximally random jammed (MRJ) state [129–131], which corresponds to packing densities in the range of *φ ≈* 0.64 depending on the applied generation protocol and on the incipient degree of ordering (crystallinity) [132,133]. The analogous problem for hard-sphere chains at very high volume fractions remained until recently elusive because of the very slow relaxation dynamics of polymeric systems resulting from the intermolecular topological constraints as explained in the introduction. As a consequence while there has been an appreciable body of simulation studies at intermediate volume fractions [134–140], most algorithms fail to provide equilibration or even generate configurations at volume fractions above the melting transition (*φ^M^* *≃* 0.545, [141]). In our approach we employ the MC scheme built around the sEB and sIEB moves described above for the generation and the successive relaxation of the hard-sphere chain configurations.

For the creation of the initial structures, we start from very dilute non-overlapping configurations (*φ ≈* 0.01) and apply the MC mixture. In more detail, it consists of the following moves, where the numbers in parentheses denote percentage attempt probabilities: (i) sEB (0.1%), (ii) sIEB (0.1%), (iii) adaptive-bias reptation (10%), (iv) adaptive-bias intermolecular reptation (25%, (v) adaptive-bias rotation (10%), vi) adaptive-bias flip (10%) and adaptive configurational bias (20%). For the generation phase, at regular intervals (every 500 MC steps) isotropic shrinkages of the simulation cell are attempted with the chains being affinely repositioned, while preserving their bonded geometry, subject to the amplitude of the box shrinkage, *dl*, along each dimension and the distance between their first monomer and the box origin [115]. The optimum value of *dl* varies strongly with volume fraction and in dimensionless form (divided by *σ*) becomes on the order of *O*(10^−7^) as we approach the MRJ state [115]. It is thus evident that by applying so small reductions in box size very long simulations, especially compared to the ones for monomeric analogs, are required in order to create dense and nearly jammed random packings of freely-jointed, hard-sphere chains. In the second phase, very long MC simulations (in the order of billions of MC steps), built around the proposed chain-connectivity altering algorithm and cast in the *nN*_ch_V Tμ* ensemble (at constant density, excluding any type of volume fluctuations), undertake the task of providing full scale equilibration of the generated random chain assemblies.

## 3. Results from Monte Carlo Simulations on Hard-Sphere Chains

**Figure 4.**
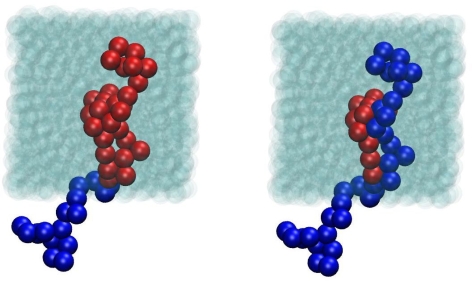
Representation of the two participating chains before (left) and after (right) the successful application of a simplified end-bridging (sEB) move from MC simulations on a *N* = 24 system at packing density *φ* = 0.63. Constituent spheres are shown with the coordinates of the centers fully unwrapped in space. Image created with the VMD visualization software [142].

**Figure 5.**
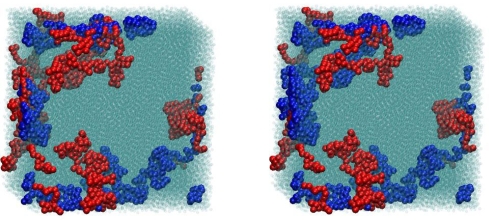
Same as in Figure 4 but for the *〈N〉* = 1000 system at volume fraction *φ* = 0.62. The coordinates of the sphere centers appear wrapped in the simulation cell subjected to periodic boundary conditions. All unmoved spheres (belonging to chains other than the participating pair) appear as transparent for visual purposes. Image created with the VMD visualization software [142].

The following systems of freely-jointed chains of tangent hard spheres have been studied: (i) 100 chains of average length *〈N〉* = 12 where chains are allowed to fluctuate uniformly in the interval *N ∈* [6*,* 18] (ii) 50 chains of *〈N〉* = 24, *N ∈* [12*,* 36] (iii) 12 chains of *〈N〉* = 250, *N ∈* [150*,* 350], (iv) 6 chains of *〈N〉* = 500, *N ∈* [300*,* 700] and (v) 64 chains of *〈N〉* = 1000, *N ∈* [600*,* 1400]. Additionally, simulations were conducted for the *〈N〉* = 12 and 24 systems with the molecular lengths following the most probable (Flory) distribution. The numerical tolerance for the identification of bond lengths was set at *dσ* = 10^−4^*σ* to guarantee maximum efficiency for the chain-connectivity altering moves at intermediate densities. Very recent simulations with strictly tangent spheres (*dσ* = 10^−8^*σ*) [120] confirm previous findings [115,143], that the allowed flexibility in bond lengths does not affect the conformational properties and local packing compared to the model of strictly tangent hard–spheres [144]. For the systems with the longer chains *〈N〉* = 250 and 500, the total simulation time ranged from 3 × 10^10^ at dilute conditions (*φ* = 0.01) to 5 × 10^11^ MC steps at densities close to the MRJ state. System configurations including sphere coordinates and chain connectivity along with MC statistics were recorded every 1 × 10^7^ resulting in simulation trajectories consisting of thousands of uncorrelated MC frames. Figure 4 shows a typical system snapshot of the two participating chains with the coordinates of the centers of the constituent sites fully unwrapped in space before and after the execution of an sEB move for the *〈N〉* = 24 system with 50 chains at *φ* = 0.63. In an analogous fashion Figure 5 depicts the two participating chains of a *〈N〉* = 1000 system at *φ* = 0.62 before and and after an sEB move, now with sphere centers subjected to periodic boundary conditions. From a brief inspection of the two figures it is immediately apparent that within a single successful sEB move profound alternations take place regarding the internal structure and connectivity of the participating chains.

**Figure 6.**
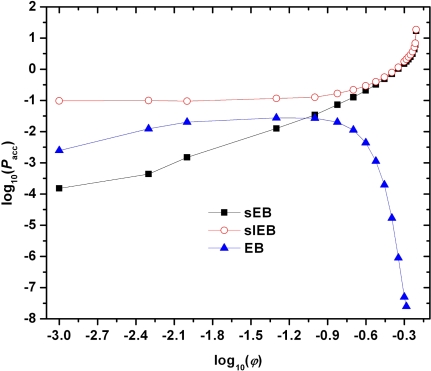
Logarithm of the percentage acceptance rates, log_10_(*P*_acc_) for the simplified end-bridging (sEB), simplified intramolecular end-bridging (sIEB) and of the original end-bridging (EB) moves as a function of the logarithm of packing density, log_10_(*φ*) as obtained from MC simulations on a *〈N〉* = 500 system of freely-jointed hard-sphere chains.

A major advantage of the set of the sEB and sIEB moves compared to the original algorithms (EB and IEB) but also to the localized algorithms steams from their particular form of application in combination with the applied hard-core potential: they are able to provide robust re-arrangements in chain connectivities through bond swapping without displacing any sites as explained in detail in sections 2.1. and 2.2.. Therefore, it is expected that the new set of moves would be tailored to function at very dense random packings, even in the close vicinity of the MRJ state. The validity of the above speculation is verified by the data shown in Figure 6 where the logarithm of the percentage acceptance rates of the sEB and sIEB moves along with the corresponding one of the original EB algorithm are plotted as a function of the logarithm of packing density for the *〈N〉* = 500 system of 6 chains.

At very low concentrations the chain-connectivity altering moves, especially the EB and sEB moves which require the close proximity of segments belonging to different macromolecules, exhibit very low acceptance rates since in a highly diluted environment chains do not “feel” the presence of each other as they are far apart. Consequently, there exist very few to zero pairs of intermolecular neighbors that lie close within the bridgeable distance of 4*σ* and *σ* + *dσ* for the EB and sEB moves, respectively. Not surprisingly, in the dilute regime the acceptance rate of sEB is the lowest since it requires the closest proximity of intermolecular neighbors. On the other hand sIEB exhibits low, but still superior compared to the intermolecular variants, acceptance rate as the probability of a chain to coil is higher than to lie close to another chain. In other words at very dilute systems a chain end finds easier intramolecular neighbors than intermolecular ones within a specified radius. We should note that the low acceptance rates of the moves at very low packing densities is not a practical problem since the corresponding acceptance rates of the localized algorithms are so high that a MC scheme built around the CCB move with deep cuts (*i.e.*, including the reconstruction of many chain monomers) is adequate to provide rapid full-scale equilibration. As packing density increases chains come closer and tend to coil leading to an increase in the acceptance rate of all moves as shown in Figure 6. At intermediate densities the efficiency of the conventional EB starts to decrease as the bridging construction and the consequent re-positioning of three spheres leads to overlaps with the unmoved spheres of the system. It is further evident that after a volume fraction of approximately *φ ≃*0.45 the acceptance rate of EB drops to such low levels (*P*_acc_ *∼ O*(10^−7^)) that the inclusion of the move in the MC scheme becomes practically fruitless. In sharp contrast, the acceptance probabilities of both sEB and sIEB get continuously augmented with increasing packing density. Given that the moves do not entail site displacements their performance depends solely on the wealth and the update rate of the lists of the initiator pairs for each move (see also related discussion in sections 2.1. and 2.2.). As density increases, and especially close to the random close packing, chains collapse in size [145] a characteristic that favors the sIEB move and come closer, almost jammed, a trend that alleviates the performance of sEB. Hence, it is not surprising the fact that both moves exhibit their highest acceptance rates at the maximally random jammed state (MRJ), in sharp contrast to all conventional MC moves [115].

**Figure 7.**
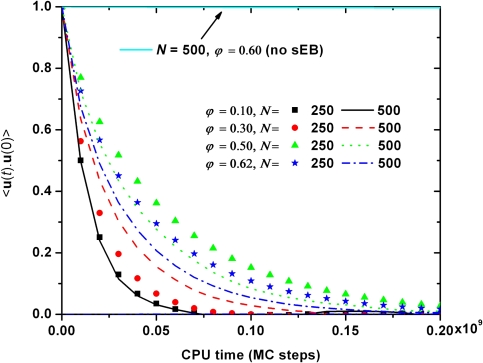
Orientational autocorrelation function of the end-to-end unit vector *〈***u**(t) · **u**(0)*〉* as a function of CPU time (in MC steps) for the *〈N〉* = 250 and 500 systems at various packing densities *φ*. Also shown for comparison purposes is the corresponding curve from MC simulations with the original EB algorithm on the *〈N〉* = 500 system at *φ* = 0.60.

While the data on the acceptance rate reveal significant limitations and advantages of the employed simulation algorithms their ability in equilibrating the long-range characteristics of polymer systems is typically quantified by the evolution in (computational) time of the orientational autocorrelation function of the end-to-end unit vector *〈***u**(*t*) · **u**(0)〉 where **u**(*t*) and **u**(0) are the end-to-end unit vectors at time *t* and reference time *t* = 0, respectively, and *〈..〉* denote averaging over all chains and MC frames by employing multiple time origins [41]. Starting from the initial value of unity the faster *〈***u**(*t*) *·* **u**(0)*〉* drops to zero the faster the system looses the memory of the its initial chain configuration. Thus, the evolution (decay) of the autocorrelation function *〈***u**(*t*) *·* **u**(0)*〉* serves as an accurate measure of the relaxation rate even if MC simulations can not provide dynamical information regarding the evolution of system in time. Figure 7 presents *〈***u**(*t*) *·* **u**(0)*〉* as a function of MC steps as obtained from MC simulations on the *〈N〉* = 250 and 500 systems at various volume fractions spanning the whole concentration range from dilute to nearly jammed packings. It is evident that packing density bears only a minor effect on the equilibration efficiency of the proposed MC algorithm as all decay curves fall very close to each other. In addition, similar trends appear regarding the effect of chain length on relaxation time: at all packing densities, within the statistical error which is relatively high because of the limited number of existing chains, there is no clear difference between the curves of the *〈N〉* = 250 and 500 systems. The performance of the MC mixture built around the sEB and sIEB moves is further compared against a conventional MC scheme that bears the original EB algorithm for the *〈N〉* = 500 system at *φ* = 0.60. As seen in Figure 7 the new MC scheme outperforms the conventional one by many orders of magnitude. Even more, as the *〈***u**(*t*) *·* **u**(0)*〉* curve for the latter does not leave the original value of unity over the whole simulation time it is perfectly clear that the system is trapped in its initial configuration with no hint of long-range equilibration.

From the time evolution of the *〈***u**(*t*) *·* **u**(0)*〉* one can calculate the total equilibration (or decorrelation) time, *τ_c_* as the integral of the form:

(7) $$\tau_{c}\;=\;\int_{0}^{\infty }\langle \text{u}(t)\;\cdot \;\text{u}(0)\rangle dt$$

**Figure 8.**
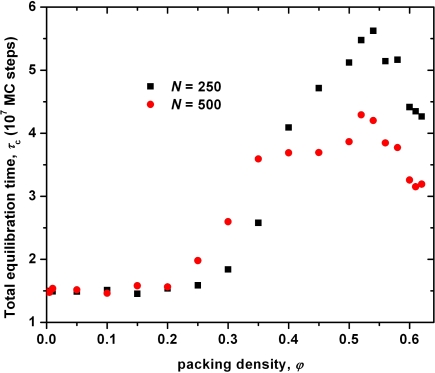
Total equilibration time *τ_c_*, versus packing density *φ*, as obtained from MC simulations on the *〈N〉* = 250 and 500 systems. The corresponding equilibration time using the original EB algorithm for the *〈N〉* = 500 system at *φ* = 0.50 is approximately *τ_c_* *≃*2500 *×*10^7^ MC steps.

Calculated values of *τ_c_* as obtained from the integration of the *〈***u**(*t*) *·* **u**(0)*〉* curves like the ones shown in Figure 7 are reported as a function of packing density for the *〈N〉* = 250 and 500 systems in Figure 8. Past MC simulations on hard-sphere systems of short chains (*〈N〉* = 12 and 24) [115] suggested that the total decorrelation time appears to be unaffected by both the average chain length and the volume fraction. Here, from the simulation data of Figure 8, we are in position to confirm the validity of the previous findings even for entangled model systems that lie deep in the polymeric regime. In particular, at intermediate and high densities (*φ >* 0.40) and especially in the vicinity of the MRJ state the longer system *〈N〉* = 500 appears to equilibrate even faster than the shorter one *〈N〉* = 250 regarding the long-range characteristics. This behavior is reminiscent of the ones of the EB [62,63] and DB algorithms [64,65,72,79,116] when applied on realistic polymeric systems in atomistic detail. Furthermore, as in the case of oligomers [115], the relaxation of random assemblies of long freely-jointed hard-sphere chains appears to be unaffected by packing density. For example, the equilibration times for the *〈N〉* = 500 system are equal to *τ_c_* *≃*3.6 and 3.2 *×*10^7^ MC steps at *φ* = 0.35 and 0.62, respectively. For comparison the corresponding time with the conventional MC scheme, where the sEB and sIEB are replaced by the original EB and IEB moves, grows to *τ_c_* *∼* 2.5 *×*10^10^ steps at *φ* = 0.50, while the analogous relaxation at *φ* = 0.60 is so slow (see also the corresponding curve at Figure 7) that no reliable estimate can be provided from extrapolations regarding *τ_c_*.

### 3.1. Analysis of the Effect of System Size on Chain Dimensions in MC Simulations

In the remaining subsection we will investigate the effect of system size on the structural properties of model polymer systems of freely-jointed hard-sphere chains. As a test case we employ extensive MC simulations on two different realizations of the *〈N〉* = 500 system: one “small” with 6 chains and one “large” with 162 chains for a total of 3000 and 81000 interacting sites, respectively. In both cases molecular lengths were allowed to fluctuate uniformly in the interval of *N ∈* [300*,* 700] under the same simulation parameters and identical mixture of MC moves at *φ* = 0.45 and 0.60. Initial configurations were generated independently so as to ensure the absence of any kind of statistical correlation between the two simulated systems. Compared to the dimensions of the simulation cell the end-to-end distance *〈R*^2^*〉*^0.5^ of chains is smaller by a factor of approximately 2 and larger by a factor of more than 1.5 for the large and the small system, respectively, at both densities. We should note that in the continuation all reported values of the end-to-end distance (and of the contour length of the primitive path, see next section) are rendered dimensionless by dividing with the collision diameter *σ* which is set equal to one. At *φ* = 0.45 for the small system the acceptance rates of both moves is 1.1% while the corresponding percentages grow to 21 and 17% for sEB and sIEB, respectively, for the large system. In an analogous fashion at *φ* = 0.60 the acceptance rates are 4.2% (sEB) and 4.8% (sIEB) and 48% (sEB) and 51% (sIEB) for the small and large systems, respectively. The low acceptance rates exhibited by both moves for the smaller boxes steam from the fact that in many cases and because of the very limited number of chains, there are no “predator-prey” pairs to initiate the moves. We should note that at *φ* = 0.60 the acceptance rates are very close to 50% as would be expected from the formulas of the acceptance criteria of the moves applied on homogeneous, isotropic chain system of adequate size.

**Figure 9.**
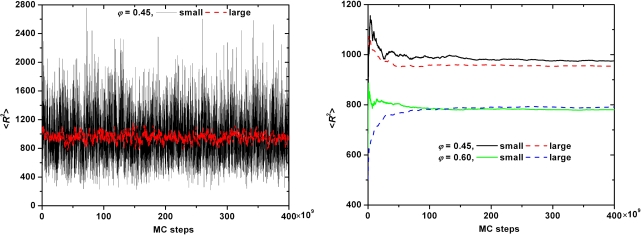
(a) Instantaneous values of the mean square end-to-end distance, *〈R*^2^*〉* as a function of MC steps as obtained from simulations on the “small” (6 chains) and “large” (162 chains) *〈N〉* = 500 hard-sphere chain system at a packing density of *φ* = 0.45. Simulation times for all systems in the x-axis are rescaled with respect to the maximum time for visualization purposes. (b) Same as in (a) but for the running average values of *〈R*^2^*〉* at *φ* = 0.45 and 0.60.

The evolution of the instantaneous value of the mean square end-to-end distance *〈R*^2^*〉* as a function of MC steps is shown in Figure 9a for the small and large systems at *φ* = 0.45 while Figure 9b shows the corresponding results for the running average of *〈R*^2^*〉* values at both densities. The calculated values are: (i) *φ* = 0.45: *〈 R*^2^*〉* = 967 (small) and 948 (large) with the relative error being less than 1.9% and (ii) *φ* = 0.60: *〈R*^2^*〉* = 778 (small) and 789 (large) with the relative error being less than 1.3%. Both relative errors are very low and definitely far below the statistical error in the calculation of the related quantity.

Identical conclusions are drawn for all measures of chain size and for the radial distribution functions in the level of individual spheres and for the whole range of concentrations even for nearly jammed structures. It is thus established that there exist no system size effects on the MC results that could potentially affect key findings regarding local packing [115,118,119,146], the numerical values of the scaling exponents for the dependence of chain size [145–147] and of the topological hindrance [146,147] on packing density for random packings of freely-jointed chains of tangent hard spheres. However we should note that the combination of the limited number of existing chains along with low acceptance rates (for the small system) and the short duration of the MC simulations (for the large system) results in a system–size effect on the shape of the applied molecular length distribution. The shape of the molecular length distribution deviates markedly from the expected one corresponding a uniform one. With increasing simulation time and for systems with an adequate number of chains the aforementioned problem diminishes.

A remaining effect of system size on the final configuration, which has to be taken into account into a more detailed analysis of our data, is the degree of polydispersity. Statistical properties of chain molecules eventually depend on the shape of the distribution of chain lengths in a rather nontrivial manner. Polydispersity does not affect those single chain statistical properties which are strictly proportional to *〈N〉*. The length of a representative primitive path (PP), and its number of entanglements, however, and to be discussed in Sec. 4., are not strictly proportional to *N*, but linear in *N*, to a good approximation. In order to value the potential effect of system size on the properties defined on the PP, one has to compare quantities defined on subsets with a narrow range of *N* values, rather than averages over the whole system.

## 4. Direct Topological Analysis of Entanglements and Primitive Paths in Polymeric Systems

### 4.1. Calculation of the Primitive Path

**Figure 10.**
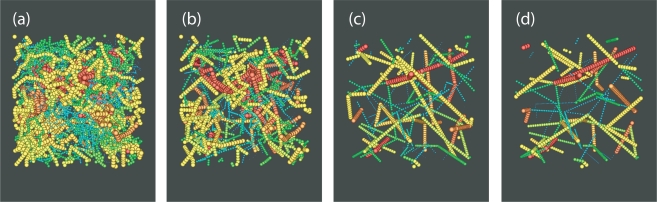
Snapshots made during runtime of the Z1 code [101,148]. (a) The original configuration, shown in folded representation, (and belonging to the set denoted as “small *φ* = 0.45” in Table 1) is (b–c) subjected to geometrical transformation which monotonously decrease the length of the multiple disconnected path until (d) the length of the path (denoted as primitive path network) has reached a minimum. The CPU time needed to complete the process stays well below 0.5 seconds.

**Figure 11.**
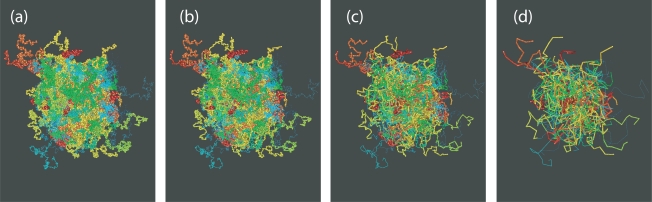
Snapshots made during runtime of the Z1 code [101,148]. (a) The original configuration, shown in unfolded representation (and belonging to the set denoted as “large *φ* = 0.60” in Table 1) is (b–c) subjected to geometrical transformation which monotonously decrease the length of the multiple disconnected path until (d) the length of the path (denoted as primitive path network) has reached a minimum. The whole operation requires about a single CPU second on a single processor.

For the melt configurations the reduction to primitive paths was performed using a procedure described in Refs. [101,106]. In this method, known as Z1 code [148], all parent atomistic (or coarse-grained) chain ends are fixed in space, cf. Figure 1. Excluded volume interactions are disabled while chain uncrossability is retained. Contour lengths of the polymeric backbones are strictly monotonically reduced through geometrical transformations. This operation leaves us with an entanglement network of so called primitive paths (see Figures 10 and 11 for a series of snapshots for selected systems in both folded and unfolded representation). Each original chain has its primitive path of length *L*_pp_, and paths have zero thickness. In addition to the set of lengths of the primitive paths, and the configuration of the entanglement network, the Z1 analysis also yields the number of interior “kinks” [101], *Z*, in the three-dimensional primitive path of each chain (see Figure 1). For long chains, *Z* is considered to be proportional to the number of entanglements, regardless of the details of the definition of entanglement. Below we are going to comment on this measure and details of the Z1 algorithm, which had not been mentioned in the literature so far. In the following it will be important to keep in mind, that while the structure and contour length of the entanglement network is a robust measure of the topological state of a polymeric system, a derived quantity like *Z* is obtained in a postprocessing step, and its definition is thus open for physically motivated refinement.

**Figure 12.**
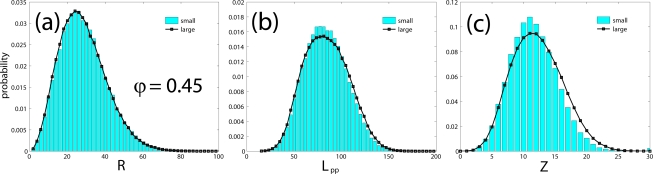
Probability distributions for various quantities resulting from the entanglement analysis (Z1) for the system at *φ* = 0.45. (a) End–to–end distance, (b) contour length of the primitive path, *L*_pp_, and (c) number of interior kinks, *Z*. This procedure constitutes the typical approach followed when analyzing monodisperse systems.

**Figure 13.**
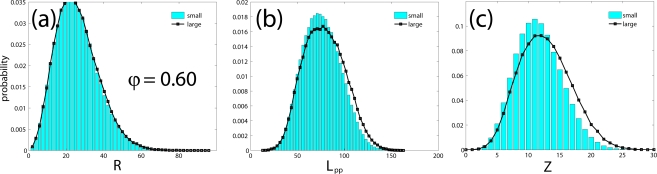
Same as Figure 12 for a larger packing fraction, φ = 0.60. For a better comparison, values range exactly as in Figure 12. Averages obtained from the distribution have been collected in Table 1.

Within the Z1 code periodic images of the same chains are treated as different chains, while all parts of a physically connected chain (which may cross the border of the simulation box) are treated as belonging to the same chain. The minimization procedure terminates as soon as the mean length of the primitive paths, *〈L*_pp_*〉*, has converged. Self entanglements are neglected in the mentioned sense, and their number is often considered small and inconsequential for polymeric systems [88] although a more detailed analysis on the role of self-entanglements may be required for very dense packings of exceedingly long chains. Finite size effects for the counting of entanglements (cf. Sec. 4.3.) can however not be prevented to set in when the chain dimension and mesh size of the PP network exceeds the box size. Structural quantities like the structure factor or the end-to-end distance are seen to be less affected, under melt conditions: See Figures 12a (*φ* = 0.45) and 13a (*φ* = 0.60) which show the probability distribution for end-to-end distances *R*, obtained from a long MC trajectory for both “small” (6 chains) and “large” (162 chains) systems. The corresponding histogram for the lengths of the primitive paths, *〈 L*_pp_*〉*, is drawn in Figures 12b and 13b. Discrepancies between small and large system are clearly visible, but remain minor, on the level of the length of the shortest path. The apparent differences can be traced back to polydispersity effects, rather than finite size effects, as we will demonstrate below.

The alternate CReTA method shares many similarities with Z1, and the conclusions reached here for Z1 analysis should apply similarly to CReTA results [104,106,111,112]. Differences between the original Primitive Path Analysis (PPA) presented in [87] and Z1 had been investigated and interpreted in [106]. Main differences concern a loss of entanglements (during PPA), the quantity to be minimized (energy versus length), and algorithmic speed (see also Table 1 for typical CPU times required for the entanglement analysis). The geometric approaches supersede PPA speeds by several orders of magnitude, and they are essentially parameter–free. All approaches return the primitive path network, in particular the length of the PP. The geometric approaches had been used to define a additional number of entanglements directly without referring to the statistics of the PP, while it had been always pointed out that the number of entanglements, albeit necessarily being based on the complete information of the primitive path, is not uniquely defined.

### 4.2. Algorithmic Details of the Z1 Code

**Figure 14.**
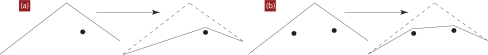
Central routine of the Z1 code finds the shortest path for two adjacent segments (solid lines) in the presence of none, one (a), two (b), or more obstacles. Each obstacle correspond a point which belongs to the contour of a different chain (not shown), and which intersects with the plane spanned by the two segments. Z1 locates potentially intersecting points efficiently, and operates at a variable number of nodes characterizing the shortest path. The central routine is iteratively applied to all pairs of adjacent segments until the multiple disconnected path has converged to a minimum length. The algorithm scales linearly with the total number of particles.

Within the Z1 algorithm the primitive path is a connected (mathematical) path of straight segments. Contour length of the multiple disconnected path is monotonically reduced by iteratively applying basic moves of the type shown in Figure 14. The physical information of this path is not the number of segments, but the shape and total contour length of the path. The algorithmic version may have 10 successive bonds at vanishing bond angle (and even vanishing length), but the physical information is the same if the 10 bonds would be replaced by a single straight bond with the total length of the 10 shorter bonds. The bond length of the algorithmic version is therefore not identical with what is commonly called a step length of the primitive path. A step length (Kuhn length [149]) is usually defined as the length of a hypothetical segment, so that the end-to-end distance of the primitive path and its contour length (which is the same in any of its representations) matches the corresponding expressions for a random walk. The primitive path does not belong to the class of ideal chains, insofar, it cannot be characterized by a single step length, in general. If one prefers to characterize it by a single step length, one has to use a statistical model (such as the random or semiflexible walk). Within the Z1 code, in order to obtain a physical path which carries information about entanglements, one removes non-physical information from the mathematical version, by disregarding nodes which do not change the direction of the path, and by disregarding (“removing”) segments of vanishing length (it is just an algorithmic detail, that vanishing means small and finite due to number precision; for the same reason one regards a bond angle as “vanishing”, if the cosine of the bending angle is above a threshold, which is 0.99 in Z1). The bond length of the resulting physical Primitive Path (PP) is thus nonzero, and its bond angles are nonzero. The remaining internal nodes had been called “interior kinks”, their number been denoted as *Z*. Such an interior kink, examples shown in Figure 1, might be identified with an entanglement, because the kink can only be displaced to the expense of an increase of the length of the entanglement network (mechanically disfavored).

**Figure 15.**
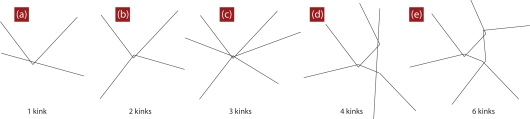
The primitive path of an individual chain is mainly characterized by its contour length *L*_pp_ and end–to–end distance *R*. The application Z1 provides not only these quantities, but information about the number of kinks. A number of possibilities are shown, where two (a,b) and three (c,d,e) chains meet, respectively. Cases (b) and (c) we call multiple entanglement events. In (a) and (d), one or more kinks meet a straight line.

Obviously, a number of kinks then corresponds to the number of changes in direction along the PP, but does not inform us on how many chains contributed to their existence, see Figure 15. All kinks are a result of at least two chains interacting, while chains can also interact without producing kinks on all (at least two) involved paths. A PP can only bend if there is at least one chain obstructing it. And in the general case there are kinks where two, three, or more chains exactly meet at a kink. Within the above mentioned picture of a step length, which requires a single chain model, this also does not matter. It matters only if we want to understand if the number of chains entangled with a given chain is larger than the number of kinks, which is possible for several physical cases, but improbable for others. First of all, PPs of several (say *x*) chains could meet in the same point in space, cf. Figure 15. If we assume that all involved PPs exhibit a kink at this point, we would have *x* interior kinks due to a multiple entanglement with *x* chains (Figure 15b–c). If all kinks were originated by twin events, and if the same chains would not exactly meet in the same point in space, but still would be all entangled, we would count 2*x* interior kinks (Figure 15e). Secondly, a straight segment can give rise to a kink on a neighboring chain, so that such an entanglement would have a single kink in total (Figure 15a), and less than 2*x* kinks, in general (Figure 15d) while a mutual entanglement, where both chains bend, has two kinks (Figure 15b). From this discussion it is clear that there is some information missing between number of interior kinks and number of entangled chains, while the former alone is used to define a step length of the PP. Because chains do not slide past each other within the Z1 code, the probability for a multiple entanglement (Figure 15c), where all chains bend at the entanglement point is expected to be small. It is simple to invent other measures, but all of them will be lacking a connection to truly topological quantities in a strict mathematical sense.

### 4.3. Comment about Self-Entanglements

**Figure 16.**
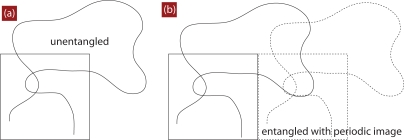
Two system size effects potentially occurring during the construction of the PP, when chain dimensions exceed box dimensions. (a) Shown is an unfolded single chain, whose ends are residing within a central simulation cell. The system is subjected to periodic boundary conditions. Because self–entanglements are excluded from the calculation of the PP, the information about connectivity has to be kept, to see, that the chain in (a) is unentan-gled. If this information is not used, self-entanglements will be underestimated, depending on system size. (b) Shown are two out of 26 cells surrounding the central cell shown in (b). Periodic images can however entangle and are treated as different chains as to minimize system size effects in the determination of the PP. Concerning the algorithm it is of importance to prevent using segments of the PP which exceed box size. The PP (not shown for this graph, cf. previous Figures 1 and 15) is thus best represented by sufficiently many, nodes, *i.e.*, more nodes than visible kinks, in Figure 1.

There is, in addition, an issue to be discussed which is relevant if the system size is small compared with the size of the chains. This issue had not been thoroughly addressed in the literature, and gives rise to confusion whenever computational resources are not sufficient to study a large enough system. As already mentioned, within the Z1 code periodic images of the same chains are treated as different chains, while all connected parts of a chain belong to the same chain, regardless of its size compared to simulation box size, see Figure 16. Self-entanglements have to be excluded from the analysis, as per original definition of the PP. It is thus important to keep information about connectivity during the PP analysis. Still, the results are prone to be biased in several respects, if chains sizes exceed system sizes, but the above choice attempts to minimize the bias. Other issues like the so–called correlation hole (see, e.g., [149]) potentially enter the discussion about self–entanglements and system–size effects.

### 4.4. Estimating the Entanglement Molecular Weight

With one of these measures, the contour length *〈L*_pp_*〉* of the primitive path, or the number of entanglements (here, *Z*), together with quantities which are already defined on the original chain, *〈N〉*, and *〈R*^2^*〉*, one can estimate an entanglement molecular weight, ideally for a monodisperse system. This quantity, denoted as *N_e_*, is defined to characterize a system composed of infinitely long chains. Unfortunately, it has always to be estimated from information about systems at finite chain length, which poses a practical problem mainly in the world of simulations.

There are remarkable and important deviations between the statistical properties of weakly entangled and infinitely long, infinitely entangled, chains, *i.e.*, between the regime where *Z* is not yet proportional to *N*, and the regime where it has approached this scaling behavior, which must ultimately happen for uniform, linear chains. These differences have been addressed recently by Hoy *et al.* in [109]. They reviewed earlier estimators and proposed new ones, for both the case where information about kinks (*Z*) is available, and the one where *N_e_* is estimated based on *〈L*_pp_*〉* and *〈R*^2^*〉*. The main findings were: (i) *N_e_* is best obtained from the linear variation of *Z* with *N*, *i.e.*, by assuming *Z* = *Z*_0_ + *N/N_e_* with two coefficients *Z*_0_ (which should be negative) and *N_e_*, the entanglement molecular weight; (ii) One has to take into account the characteristic ratio *C_N_* when estimating *N_e_* using *L*_pp_ values. The entanglement molecular weight appears here because it is defined to be the ratio between *〈N〉* and *〈Z〉*, in the limit of large *〈N〉*. The quantity *Z*_0_ reflects the fact, that a minimum amount of material is required to produce a single entanglement. We expect it to depend on the molecular details of the model and state variables such as temperature, which influence, e.g. the flexibility of chains. Upon applying the proposed procedure, we can evaluate a corrected value for *Z*, denoted as Z^*^, which is free from both polydispersity effects and effects of finite chain length via

(8) $$Z^{*}\;=\;\langle N\rangle \frac{d}{dN}\langle Z(N)\rangle$$

**Figure 17.**
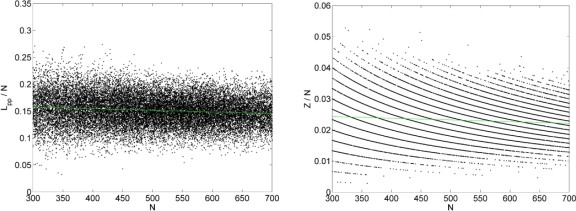
Ratios *L*_pp_*/N* (left) and *Z/N* (right) vs. *N*, for the tangent hard sphere system at *φ* = 0.60 (small system size, 6 chains). Each point is obtained from data for a single chain of chain length *N*, during the MC. While both quantities are roughly constants, there are important *o*(*N^–^*^1^) corrections [109] which lead to differences in the entanglement molecular weights and quantities listed in Table 1. The variance of *L*_pp_*/N* tends to decrease with increasing *N*, and importantly, *L*_pp_*/N* weakly decreases with *N* . Same for *Z/N*, but in addition, points have to fall onto lines because the number of kinks, *Z*, for an individual chain, is an integer. The thin straight lines result from linear regression which allows to determine a polydispersity-corrected number of kinks, and corresponding entanglement molecular weight, cf. Equations (8) and (9).

Here, the averages are calculated using all pairs of {*Z, N*} values for the individual chains (Figure 17 shows representative data). The quantity *〈Z*(*N*)*〉* is obtained using *Z* values for the subsystem of chains of size *N*, and thus depends on *N*. The derivative becomes independent on chain length when *〈N〉* exceeds *N_e_*. For the systems investigated here, this is clearly the case. Thus, Z^*^ is proportional to *〈N〉* and characterizes the entangled state of the polymeric system. This quantity can be used to estimate finite size effects of the MC generation procedure. With Z^*^ at hand we can immediately calculate an entanglement molecular weight, *N_e_*, because it is defined as the ratio between *N* and *Z* in the limit of large *N*. We thus have

(9) $$N_{e}^{*}\;=\;\frac{\langle N\rangle }{Z^{*}}$$

with Z^*^ obtained via Equation (8) from the simulation data. Values Z^*^ and $N_{e}^{*}$ are listed in Table 1. As is obvious from this table, while polydispersity effects seem to play a minor role in the large system, the correction is important for the small one. Because the values for the small systems sandwich the ones for the large system, and because the difference between the values for small and large systems is within errors, we do not detect any measurable finite size effect. An independent estimate of the entanglement molecular weight can be based on *〈L*_pp_*〉* rather than *〈Z〉*, cf. [109].

## 5. Conclusions

We have reviewed the salient methodological characteristics of an hierarchical modeling scheme for the molecular simulation of model polymer systems, and mentioned open questions. The present approach combines Monte Carlo simulations, based on chain-connectivity altering moves that undertake the task of system generation and equilibration and a direct geometrical algorithm that renders the parent polymer configurations into a primitive path mesh and fully identifies the topological constraints (entanglements) between different chains. While the proposed algorithmic approach is general and can be applied to any polymer system irrespective of the chemical constitution, repeat units or molecular architecture, we have placed particular emphasis on the application on model random packings of linear freely-jointed chains of tangent hard spheres of uniform size. We have clearly demonstrated that the MC-based long-range equilibration of these systems is affected by neither the molecular length of the chain nor by the volume fraction (packing density). In addition, we have analyzed the effect of system size in key structural properties like chain size, contour length of the primitive path, number of entanglements and entanglement molecular weight. Finally, we have commented about the issue of treating self-entanglements in the analysis, and the difficulties which prevent us from identifying entanglements as (a fixed number of) kinks of the primitive path, while the underlying goal is to avoid any assumptions about the statistics of the primitive path. Correlations between segment vectors along the PP rather than a number of kinks can be used to characterize the properties of the PP as returned by the Z1 code, but as long as the measured correlations produce a single number (like a persistence length), this number should be related to a number of kinks. We have demonstrated how, starting from state-of-the-art simulation algorithms for atomistic, chemically-simple polymer melts, we can arrive, mainly through simplifications of the original implementation, on new moves tailored to provide rapid and robust equilibration in the simulation of model random packings of hard-sphere chains. Through extensive Monte Carlo simulations based of the sEB and sIEB moves [115], reaching the order of trillions (*O*(10^12^)) of steps, we were able to identify the maximally random jammed (MRJ) state for model polymer systems and show that hard-sphere chains can be as efficiently and as densely packed as the monoatomic analogs do [118]. Additionally, the local structure and packing of the chain assemblies were studied and analyzed as a function of packing density and compared against the ones of monomeric hard-sphere systems [119,120]. In combination with the introduction and employment of new metrics [154] for the determination of local order (crystallinity) we studied the spontaneous entropy-driven disorder-order transition in dense systems of athermal chain molecules [155]. By extending the MC simulations to long macromolecular systems the dependence of chain size on packing density was studied and the characteristic scaling regimes (dilute [8,156,157], semi-dilute [8,156,157], marginal [145,156] and concentrated [8,145,156,157]) along with the corresponding scaling exponents were identified and compared against theoretical expectations in the whole density range [145]. Finally, through the combination of the equilibrated MC trajectories with advanced topological algorithms for the extraction of entanglements [101,102] and knots [158–160] we analyzed the effect of concentration on topological hindrance that steams either from intramolecular (knots) or intermolecular (entanglements) constraints [146,147].

The reviewed methodology can be applied to follow the dynamics of the primitive path, and to study the fluctuations of the primitive path characteristics, in order to extract quantities such as the tube survival probability and shear modulus [112,150–153]. The efficient calculation of the entanglement network further allows to build Monte Carlo algorithms devoted to create polymeric system with given entanglement characteristics, by tuning, e.g., density, stiffness or architecture of the polymers. The outlined methodology will also be required to set up a so called “beyond equilibrium molecular dynamics” (BEMD) algorithm, which is devoted to self-consistently obtain the “building blocks” (Poisson bracket, entropy gradient, friction matrix, cf. [102,161,162,164]) of the time evolution equation for some slow, relevant system variables. For polymer melts, these variables might involve properties such as contour length, tube diameter, stiffness, or number of interior kinks of the entanglement network [25,113,165,166]. So far the BEMD strategy has only been demonstrated for rarefied gases [102,163] and unentangled polymer melts [40].

Current efforts and future applications further include the molecular modeling of polymer samples of varied molecular architecture in the bulk and at nano-interfaces at various descriptions of detail. The proposed hierarchical scheme for the simulation of the hard-sphere chains is being presently expanded so as to include dynamical and rheological information at and beyond equilibrium.

## Figures and Tables

**Table 1.** Comparison between results for small and large systems (with same *〈N〉*) at two packing densities *φ*. Results denoted as *〈Z〉* (interior kinks) and *〈L*_pp_*〉* (contour length of the PP), *i.e.*, without a prime, are based on classical procedure for monodisperse melts. The entanglement molecular weight is evaluated via *N_e_* = *〈N 〉/〈Z〉*, cf. [109] for a discussion. We report CPU times (on a modern, conventional laptop) required to analyze a single configuration. Differences between small and large systems we have carefully traced back to be caused by the different polydispersity between small and large systems. The corrected quantities, denoted with a prime, have been evaluated via Eqs. (8) and (9), Z^*^ = *〈N〉* (*d/dN*)*〈Z/N〉* and $N_{e}^{*}\;=\;\langle N\rangle /Z^{*}$, respectively, where individual {*N, Z*} values for each chain enter, rather than their system averages. As is obvious from the table, while polydispersity effects seem to play a minor role in the large system, the correction is important for the small one. Because the values for the small systems sandwich the ones for the large system, and because the difference between the values for small and large systems is within errors, we do not detect any measurable finite size effect. An independent estimate of the entanglement molecular weight can be based on *〈L*_pp_*〉* rather than *〈Z〉* or Z^*^, cf. [109].

system	chains	*〈N〉*	*〈R*^2^*〉*^1^*^/^*^2^	CPU time	*〈L*_pp_*〉*	*〈Z〉*	Z^*^	*N_e_*	$N_{e}^{*}$
*φ* = 0.45									
small	6	500	31.1	0.12 s	83.5	11.8	13.2	42.4	37.9
large	162	500	30.8	7.93 s	84.0	12.2	12.2	40.9	40.9

*φ* = 0.60									
small	6	500	27.9	0.16 s	75.5	11.6	13.0	43.1	38.5
large	162	500	28.1	8.34 s	78.3	12.6	12.6	39.7	39.7
